# Implementation of a Virtual Reality Intervention in Outpatient Physiotherapy for Chronic Pain: Protocol for a Pilot Implementation Study

**DOI:** 10.2196/58089

**Published:** 2024-09-23

**Authors:** Alexander Elser, Christian Kopkow, Axel Georg Schäfer

**Affiliations:** 1 Faculty of Social Work and Health HAWK University of Applied Sciences and Arts Hildesheim/Holzminden/Göttingen Hildesheim Germany; 2 Faculty 4 for Human Sciences Department Therapy Science I Brandenburg University of Technology Cottbus - Senftenberg Cottbus - Senftenberg Germany

**Keywords:** chronic pain, implementation, virtual reality, VR, physiotherapy, virtual reality intervention, pain, outpatient, chronic pain conditions, evidence-based

## Abstract

**Background:**

Chronic pain is a global health issue that causes physical, psychological, and social disabilities for patients, as well as high costs for societies. Virtual reality (VR) is a new treatment that provides an opportunity to narrow the gap between clinical practice and recommended care in the use of patient education and behavioral interventions in the outpatient physiotherapy setting. However, there is currently no implementation strategy to integrate VR treatments into this setting.

**Objective:**

This protocol outlines a pilot implementation study that aims to (1) identify barriers and facilitators for implementing a VR intervention in outpatient physiotherapy care for people with chronic pain and (2) develop and pilot test an implementation strategy in 5 practices in Germany.

**Methods:**

The study consists of 4 phases. The first phase involves adapting the treatment protocol of the VR intervention to the local context of outpatient physiotherapy practices in Germany. The second phase includes the collection of barriers and facilitators through semistructured interviews from physiotherapists and the development of a theory-driven implementation strategy based on the Theoretical Domains framework and the Behavior Change Wheel. This strategy will be applied in the third phase, which will also include a 6-month span of using VR interventions in practices, along with a process evaluation. The fourth phase consists of semistructured interviews to evaluate the developed implementation strategy.

**Results:**

The recruitment process and phase 1, including the adaptation of the treatment protocol, have already been completed. We recruited 5 physiotherapy practices in Lower Saxony, Germany, where the VR intervention will be implemented. The collection of barriers and facilitators through semistructured interviews is scheduled to begin in February 2024.

**Conclusions:**

This pilot implementation study aims to develop a theory-driven implementation strategy for integrating a VR intervention into outpatient physiotherapy care for people with chronic pain. The identified barriers and facilitators, along with the implementation strategy, will serve as a starting point for future randomized controlled implementation studies in different settings to refine the implementation process and integrate VR interventions into the outpatient care of people with chronic pain.

**Trial Registration:**

German Clinical Trials Register DRKS00030862; https://tinyurl.com/3zf7uujx

**International Registered Report Identifier (IRRID):**

DERR1-10.2196/58089

## Introduction

Chronic pain defined as persistent or recurrent pain lasting longer than 3 months [1] is a major global health problem. Chronic pain conditions (back pain, musculoskeletal disorders, and neck pain) are 3 of the leading causes for years lost to disability in the last decades [2,3]. Prevalence rates in industrial nations such as the United States (20.5%) [4], Germany (28.3%) [5], the United Kingdom (34%) [6] and Chile (48.1%) [7] are high, but also among low- and middle-income countries which prevalence rates ranging from 13% to 49.4% [8]. Chronic pain often results in physical disability, psychological distress, and reduced quality of life [3,9], and is associated with higher rates of divorce and suicide, and affects relationships and self-esteem [10,11]. For societies, chronic pain is an enormous financial burden on the health care system. The financial cost of chronic pain in the United States is estimated to be between US $560 billion and US $635 billion in 2010, including medical costs and lost productivity [12]. In Germany chronic pain costs at least €53.9 billion (US $59.4 billion) annually [13]. Although evidence-based treatments and guidelines for the management of chronic pain are available [14-16], they are only partially used [17,18]. In Germany, for example, only 38% of physiotherapists in a survey reported that they work according to guidelines in chronic pain management [19]. Perceived barriers to the use of guidelines in clinical practice include lack of time, lack of research skills, limited access to guidelines, and inapplicability of guideline recommendations to individual patients [19,20].

Implementation studies offer an opportunity to improve this situation and overcome these barriers by developing and testing targeted implementation strategies for evidence-based interventions [21]. Currently, frameworks, models and theories for the development of implementation interventions and strategies are not yet used as standard in the implementation of virtual reality (VR) interventions, although this is recommended. [22]. One model to guide the process of implementation is the knowledge to action (KTA) cycle [23]. The KTA cycle is a process model, which is increasingly used since its introduction in 2006 [24-27]. The KTA was developed from a concept analysis of 31 planned action theories to make sense of the complex implementation process. It provides a comprehensive view by integrating concepts of knowledge creation and application. At each stage of the action cycle, other frameworks or theories can be applied [23].

One such framework to determine barriers and facilitators is the Theoretical Domains framework (TDF) [28,29]. The TDF was created to enhance health care researchers’ access to psychological theory by providing a systematic and simplified approach to behavior change theories. Consisting of 14 theoretical domains derived from 33 theories and 128 constructs, this framework serves as a valuable tool for examining the barriers and facilitators that influence professional behavior change. Once the barriers and facilitators in a setting have been identified, they can be mapped to the domains. The behavior change wheel (BCW) [30] can then be used to develop implementation interventions with specific functions and behavior change techniques to influence the targeted behavior. The BCW is a comprehensive framework that incorporates behavioral theory to effectively capture and address the mechanisms of action within interventions. Developed through expert consensus and a rigorous validation process, the wheel is organized into 3 levels. Its central element is the capability, opportunity, and motivation model of behavior change (COM-B model), which includes aspects of capability (both physical and psychological), opportunity (both social and physical), and motivation (both automatic and reflective). It is proposed that people need these 3 factors to increase the likelihood of performing the behavior in question [30].

A new evidence-based intervention for people with chronic pain is VR [31]. It is used in physiotherapy in many specialties such as neurology and orthopedics to manage pain [32]. VR treatments include VR games, mindfulness-based interventions, practical exercises, and visual illusions for people with chronic pain [33]. For example, 1 part of an intervention is a relaxation exercise in which deep breathing is used to make a virtual tree blossom. Another is a virtual journey through the body to explain chronic pain to the patient. A meta-analysis showed large effects of VR interventions on pain (standardized mean difference [SMD] 1.6, 95% CI 0.83-2.36) and body functioning (SMD 1.4, 95% CI 0.13-2.67) in people with chronic pain [33]. However, it is unknown how often VR treatments are used in the care of people with chronic pain. A recent review about the barriers and facilitators in the implementation of VR intervention for people with chronic pain shows various barriers. The main barriers were the inappropriate choice of VR devices and VR interventions in relation to the patients, and that patients did not have the required skills to use VR and no tutorials or instructions were available [34]. Though, there are also some facilitators to the implementation of VR interventions for people with chronic pain, such as positive beliefs about consequences from the patient's perspective [34]. However, little is known regarding barriers and facilitators from a health professional perspective. The next step in the implementation of VR interventions into health care is the development of an initial implementation strategy, supported by implementation frameworks focusing on behavior change of all stakeholders [35]. Therefore, the aim of this study is (1) to identify barriers and facilitators from the perspective of health care professionals and (2) to develop and test a strategy for the implementation of VR interventions in outpatient physiotherapy practices.

## Methods

### Overview

**Figure 1:**
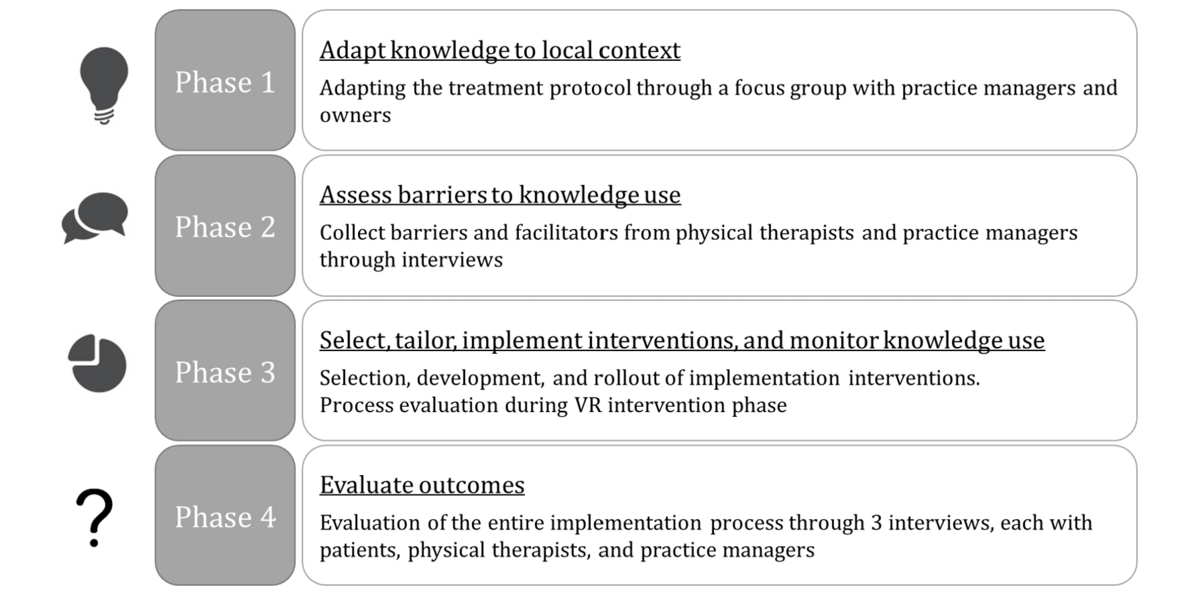
The four phases of the pilot implementation study for the development and evaluation of an implementation strategy. VR: Virtual reality.

The 4 phases (Figure 1) of the pilot implementation study are derived from the KTA cycle [23] and the entire implementation process is accompanied by a formative evaluation [36], which makes it possible to collect data at several points throughout all phases and influence the implementation at the same time. The implementation takes place in 5 outpatient physiotherapy practices in Germany. In the first phase (adapt knowledge to local context), the treatment protocol of the VR intervention is adapted to the context of outpatient physiotherapy practices. The second phase (assess barriers to knowledge use) consists of collection of specific barriers and facilitators to the implementation of the VR intervention from a health care professional perspective. Participating therapists first receive training in the use of the VR devices and the VR intervention, and then semistructured interviews are conducted with them. This will be the basis for the development of an implementation strategy with tailored implementation interventions in phase 3 (select, tailor, implement interventions, and monitor knowledge use). After the implementation strategy has been carried out, the physiotherapists will use the VR intervention in the practice with an accompanying process evaluation. Finally, the entire process and implementation strategy will be evaluated and adapted in phase 4 (evaluate outcomes).

### VR Intervention

**Figure 2:**
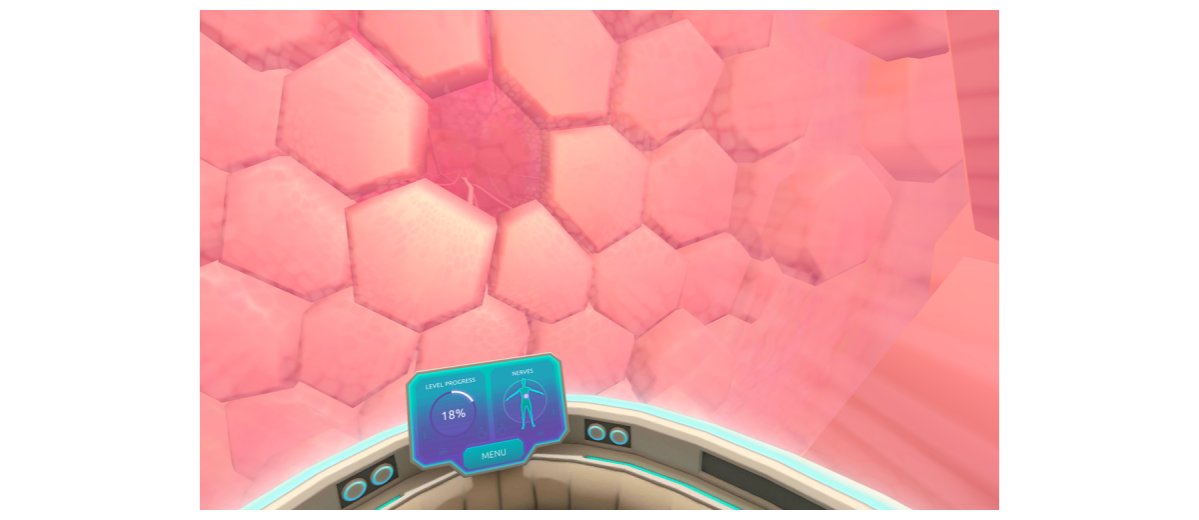
Screenshot from the nerve level of Reducept.

The VR intervention (Reducept, the Netherlands) is a psychological VR intervention for the treatment of chronic pain. Reducept aims to teach patients that pain can be influenced and managed by changing the way they think about their pain. In the game, the user travels through the body in a spaceship (Figure 2). Along the way, the user learns about the mechanisms of pain. Reducept is based on the “explain pain” guidelines [37], which state that understanding pain and influencing the cognitive, emotional, and behavioral processes associated with it enables patients to reduce their pain experience. In addition to pain education, the user plays several serious games that stimulate the visual, auditory, and proprioceptive systems to control chronic pain through distraction and relaxation. The VR intervention uses techniques from cognitive behavioral therapy, acceptance and commitment therapy, mindfulness, and hypnotherapy [38]. The game is divided into 5 modules that build on each other and last from 5 to 10 minutes. A workbook with in-depth content on the VR modules accompanies the therapy. The planned therapy sequence consists of 6 therapy sessions of 10 to 20 minutes each.

**Data Analysis**

Qualitative content analysis according to Kuckartz [39] will be used throughout the study to analyze qualitative data. Qualitative content analysis is a systematic and structured way of analyzing qualitative data. First, main categories are identified deductively or inductively, then text segments are systematically assigned to these categories. From these text segments, subcategories are inductively derived. This method helps to identify themes, patterns, and correlations in the data, thus providing deeper insights into the phenomenon under study. In addition, Kuckartz content analysis provides a transparent approach that increases the validity and reliability of research findings. MAX Qualitative Data Analysis (MAXQDA Plus 2022, version 22.0.1, VERBI software) will be used for the qualitative analysis.

The data related to the patients’ health outcomes are evaluated for normal distribution and sphericity, and then analyzed using repeated measures ANOVA in SPSS (version 28.0.1.0, IBM Corp).

### Phase 1

The first phase of the implementation process consists of adapting the treatment protocol of the VR intervention to the context of outpatient physiotherapy practices in Germany.

In total, 5 outpatient physiotherapy practices in Lower Saxony (Germany) will participate in the study and implement the VR intervention. At least 5 physiotherapists must work in the practices to be eligible to participate in the study. Practice managers or owners and 2 researchers will participate in guided focus groups to adapt the original treatment protocol of the VR intervention. Focus groups are a good method to investigate what participants think and why they think it [40]. In addition, the group setting can stimulate new ideas that may be hidden in individual discussions [41]. Another goal is to establish collaboration and networking among participants. The focus group guideline will be developed using the implementation outcomes of acceptability, appropriateness, adoption and feasibility [42]. The guideline will be pretested and adapted, if necessary.

The focus group will take place at the University of Applied Science and Arts in Hildesheim/Holzminden/Göttingen (Germany) and will last approximately 2 hours. One researcher will moderate the focus group, while a second researcher will take care of technical aspects, keep time, and take notes on important aspects or special incidents.

At the beginning of the focus group, the moderator gives an introduction explaining the process and goals of the overall study and the focus group, followed by a presentation of the original VR treatment protocol. During the focus group, a table is displayed with the content and duration of the 6 VR treatment sessions originally planned in the treatment protocol. The main part of the focus group addresses the 4 outcomes of acceptability, appropriateness, adoption, and feasibility in relation to the treatment protocol. By discussing these aspects, it will be possible to adapt the treatment protocol to the context of outpatient physiotherapy practices. The final section is an open-ended question and answer session where participants can speak and discuss freely about the treatment protocol and possible adaptations. At the end of the focus group, participants will complete an online demographic survey. The audio of the focus group is recorded with an audio recorder for the entire duration.

In this phase, the qualitative content analysis according to Kuckartz and Rädiker [39] will focus on possible adjustments to the original treatment protocol in relation to the setting. After the analysis, the VR treatment protocol will be adapted in consultation with the practice owners to develop a treatment protocol for implementation.

### Phase 2

The second phase is derived from the “assess barriers to knowledge use” step of the KTA cycle [23]. A total of 2 physiotherapists from each practice will participate in the study. They must have completed their physiotherapy training and be employed in the practice. Participants will receive training in the use of the VR intervention, including the use of VR devices (Pico 4 [ByteDance]), as the first part of the implementation strategy. In addition, participants will receive information on how the VR intervention should be applied to people with chronic pain. This information will also be made available on demand on an online platform. After the VR training, the acceptance, appropriateness, and feasibility of the VR intervention from the therapists’ perspective will be assessed through an online survey using the German versions of the Acceptability of Intervention Measure, Intervention Appropriateness Measure, and the Feasibility of Intervention Measure questionnaires [43,44]. Then they receive the VR devices to test the intervention at home. Approximately 2 weeks later, an optional online meeting will be offered to therapists to answer any remaining questions about the VR intervention or the use of the VR devices. The barriers identified in the focus group and in the online survey after the VR training will be used to define the behaviors that need to be changed or implemented when the VR intervention is used in practices. From these behaviors, the study team will select 1 or 2 core behaviors to change that appear promising to support implementation of the VR intervention. The selection of the target behaviors will be based on the potential impact on the implementation of the VR intervention, the potential multiplier effect in practices, and the feasibility of measuring behavior change. These target behaviors are specified by the question: who needs to do what differently, when, where, how, and with whom [45]?

Approximately 1 week after the online meeting, semistructured interviews will be conducted with the participating physiotherapists to identify and specify barriers and facilitators to the implementation of the VR intervention. An interview guideline will be developed to understand the factors that may influence the implementation of VR interventions in practices. It will be based on the previously defined target behaviors and the domains of the TDF [28,29]. Before being used, the interview guide is pilot tested with 2 physiotherapists to test its comprehensibility.

The interviews are expected to last approximately 45 to 60 minutes and will be conducted online through Zoom (Zoom Video Communications). Participants will receive written informed consent before participation in the study. Interviews will be audio-recorded and transcribed verbatim. Data collection and storage will be pseudonymized. The data of the focus group and the semistructured interviews will be analyzed at the end of this phase using qualitative content analysis according to Kuckartz [39]. The TDF domains will be used as deductive codes to which the data will be assigned, to gather barriers and facilitators from the focus group and the semistructured interviews.

### Phase 3

Based on the qualitative and quantitative assessed barriers and facilitators in phase 1 and 2 and the barriers and facilitators found in a scoping review [34], an implementation strategy, consisting of various implementation interventions, will be developed. The identified domains of the TDF are mapped to the COM-B model of the BCW [46]. This helps to determine what underlying factors need to be changed in order for the therapist’s behavior to change. Based on these factors, the functions of the implementation interventions and the recommended behavior change techniques can be specified and preliminary implementation interventions (eg, training, modeling, and environmental restructuring) can be developed [46]. Affordability, practicality, effectiveness and cost-effectiveness, acceptability, side effects or safety, and equity will be used to select the most appropriate and effective implementation interventions for the implementation strategy [46]. In our study, we will not include policy categories such as regulation, fiscal and legislation, as recommended by the BCW framework. This exclusion is due to the limited timeframe and scope of our project, which does not extend to policy-level changes.

The developed implementation strategy will be discussed with the stakeholders from the participating practices, to specify the execution of the implementation interventions. The strategy will then be executed in all 5 practices and process evaluated through an online questionnaire. The strategy will be reported according to the standards of Proctor et al [47].

Patients are eligible if they are aged 18 years or older, self-report that they have a chronic pain condition, and are able to understand and speak the German language fluently. Patients will be excluded if they have a current or previous diagnosis of epilepsy, dementia, migraine, or other neurological conditions that may prevent the use of VR or lead to adverse effects such as hypersensitivity to flashing lights or severe hearing impairment. Pain intensity, disability due to pain, fear of movement, pain catastrophizing, quality of life, and pain-related self-efficacy will be assessed using the German versions of the Numeric Pain Rating Scale [48,49], Pain Disability Index [50], Tampa Scale for Kinesiophobia [51], Pain Catastrophizing Scale [52], EQ-5D-5L (quality of life) [53], and a questionnaire assessing pain-related self-efficacy [54] before and after the VR intervention.

Physiotherapists will complete an online questionnaire to document each session of the VR intervention. The questionnaire consists of questions about the fidelity of treatment delivery based on the fidelity protocol in the study by Toomey et al [55] (eg, did the therapist adhere to the treatment protocol or which components of the treatment were delivered) and whether there were any adverse events during that session. The therapists will use the VR intervention in their practices for 6 months. Before participation in the study, written informed consent will be obtained from participants. The collected process evaluation data will be analyzed descriptively using SPSS.

### Phase 4

The final phase will be an evaluation of the overall implementation process and an analysis of the health-related outcomes of the patients.

The evaluation of the implementation process will be done through semistructured interviews with 3 patients, 3 therapists, and 3 practice owners. Patients must have received the full treatment duration to be eligible for the interviews, therapists are included if they have treated at least 5 patients with Reducept, and 3 participants are selected from the practice owners of the participating practices. Before the interviews, all participants will complete the Acceptability of Intervention Measure, Intervention Appropriateness Measure, and the Feasibility of Intervention Measure questionnaires. The data from the questionnaires, noticeable aspects from the process evaluation in phase 3 and the implementation outcomes adoption, acceptability, appropriateness, feasibility, fidelity, and implementation costs [42] will be used to develop an interview guide. The interviews will determine the perceived effectiveness of the implementation strategy was, how the VR intervention was used in the practices, how patients perceived the use of the VR intervention and give indications how the implementation strategy can be adapted for future projects. Before use, the interview guide is pilot-tested with people from the same background who are not part of the study to ensure comprehensibility.

The interviews will be conducted online through Zoom and are expected to last approximately 60 minutes. Written informed consent will be obtained from participants before participation in the study. Interviews will be audio-recorded and transcribed verbatim. Data collection and storage will be pseudonymized. Data will be analyzed using Kuckartz’s qualitative content analysis [39].

This data will be used to adapt the implementation strategy and to create recommendations for the implementation of VR interventions for people with chronic pain in outpatient physiotherapy practices.

### Ethical Considerations

Ethical approval for the study has been obtained from the ethics committee of the University of Applied Science and Art in Hildesheim/Holzminden/Göttingen (Germany) (April 3, 2023). The principles of the Declaration of Helsinki [56] will be strictly followed in this research project. Eligible participants will be informed of the objectives and procedures of the study. Before inclusion, written informed consent will be obtained from all patients. Participation will be voluntary, and participants will be free to withdraw from the study at any time. The reporting of this protocol follows the CONSORT (Consolidated Standards of Reporting Trials) 2010 statement: extension to randomized pilot and feasibility trials [57]. The study is registered in the German Clinical Trials Register (DRKS00030862).

## Results

The recruitment process and phase 1 with the adaption of the treatment protocol are already conducted. In total, 5 physiotherapy practices were identified in which the VR intervention is to be implemented. A focus group with 4 practice owners was held in November 2023. Due to personal appointments, 1 owner had to cancel the focus group at short notice and was subsequently interviewed by a semistructured interview about the treatment protocol. The data was then analyzed by qualitative content analysis and used to adapt the treatment protocol.

## Discussion

### Principal Findings

The described pilot implementation study will provide insight into the implementation of VR interventions for people with chronic pain managed in outpatient physiotherapy practices. The developed implementation interventions and implementation strategy can be foundation for future implementation projects. Especially the insights into the perspective of health care professionals will play an important role in the further implementation of VR interventions for people with chronic pain, as they play an important role in the implementation of digital interventions [58]. The implementation of VR interventions can lead to better outpatient care for people with chronic pain, as VR can help reduce pain, positively influence disability, and reduce fear of movement [59-61].

Previous research suggests that technology adoption in health care is still lacking due to technically challenged staff, resistance to change, high costs and low acceptance [34,62,63]. In the implementation of VR in the organizational structures and VR technology itself have been barriers to the implementation of VR interventions in various health care settings [35,64,65]. Regarding the use of VR in physiotherapy, VR itself seems to be the main barrier due to technical issues, lack of guidelines for VR interventions, and patient-related factors [32]. In contrast, staff and health care professionals can act as facilitators by reducing anxiety about new technologies and changing attitudes toward VR [32,35,65]. There is also a general interest in the use of VR in rehabilitation among health care professionals [35]. The development and application of systematic implementation strategies can help to promote the use of new technologies and VR. By identifying the barriers and facilitators in each setting, it will be possible to target implementation interventions to change the behavior of health care professionals, patients, and practice owners. The implementation study described here can develop an initial implementation strategy for the implementation of VR interventions for the outpatient physiotherapy treatment of people with chronic pain. This strategy can be a first step to increase the acceptance and use of VR interventions in the field of chronic pain, leading to better patient care. The next step in implementing VR interventions in physiotherapy practices should be to evaluate the effectiveness of the implementation strategy in a controlled trial.

### Limitations

This study has some limitations that should be noted. First, the lack of a control condition (eg, cluster randomization with other physiotherapy practices) makes it difficult to accurately assess the effectiveness of the implementation strategy. Second, the small sample size of only 5 practices limits the generalizability of the implementation strategy. In addition, involving all stakeholders in each phase of the study may allow for more comprehensive input into the development of implementation interventions.

### Conclusions

The findings of this pilot implementation study will contribute to the body of knowledge regarding the development process and effectiveness of an implementation strategy for VR interventions for the treatment of people with chronic pain in outpatient physiotherapy practice. The objective is to develop an initial implementation strategy that can be used in future implementations of VR, with adaptation if necessary. The successful implementation of VR interventions in the outpatient treatment of people with chronic pain will lead to increased guideline adherence among physiotherapists and therefore to better care.

## Abbreviations

BCW: behavior change wheel
COM-B: capability, opportunity, and motivation model of behavior change
KTA: knowledge to action
SMD: standardized mean difference
TDF: Theoretical Domains framework
VR: virtual reality

## Data Availability

The data sets generated during and analyzed during this study are available from the corresponding author on reasonable request.
